# Mitochondrial Genome Assembly and Structural Characteristics Analysis of *Gentiana rigescens*

**DOI:** 10.3390/ijms252111428

**Published:** 2024-10-24

**Authors:** Zongyi Xie, Yingmin Zhang, Lixin Wu, Guodong Li

**Affiliations:** 1 College of Chinese Medicine, Yunnan University of Chinese Medicine, Kunming 650500, China; 2 Yunnan Key Laboratory of Dai and Yi Medicines, Yunnan University of Chinese Medicine, Kunming 650500, China

**Keywords:** *Gentiana rigescens*, mitogenome, multichromosomal genome, intracellular gene transfer

## Abstract

*Gentiana rigescens*, an alpine plant with significant medicinal value, possesses a complex genetic background. However, comprehensive genomic research on *G. rigescens* is still lacking, particularly concerning its organelle genome. In this study, *G. rigescens* was studied to sequence the mitochondrial genome (mitogenome) and ascertain the assembly, informational content, and developmental expression of the mitogenome. The mitogenome of *G. rigescens* was 393,595 bp in length and comprised four circular chromosomes ranging in size from 6646 bp to 362,358 bp. The GC content was 43.73%. The mitogenome featured 30 distinct protein-coding genes, 26 tRNA genes, and 3 rRNA genes. The mitogenome of *G. rigescens* also revealed 70 SSRs, which were mostly tetra-nucleotides. In addition, 48 homologous fragments were found between the mitogenome and the chloroplast genome, with the longest measuring 23,330 bp. The documentation of the mitochondrial genome of *G. rigescens* is instrumental in advancing the understanding of its physiological development. Decoding the *G. rigescens* mitogenome will offer valuable genetic material for phylogenetic research on Gentianaceae and enhance the use of species germplasm resources.

## 1. Introduction

*Gentiana rigescens*, a perennial herbaceous plant of the Gentianaceae family and the genus *Gentiana*, thrives in the diverse ecosystems of southwestern China, including Yunnan, Sichuan, and Guizhou provinces. Typically found in meadows, shrublands, valleys, and beneath forest canopies, this resilient species adapts to altitudes between 1100 and 3000 meters. Recognized as one of the original sources of “Longdan” in the Chinese Pharmacopoeia [1], *G. rigescens* holds a special place in traditional Chinese medicine. The Yi nationality, one of China’s ethnic minorities, has long utilized “Longdan” for its therapeutic properties, particularly in treating sore throats and urinary tract infections.

Mitochondria are indispensable organelles in eukaryotic cells, serving as a crucial site for respiration and energy conversion [2]. Mitochondria are genetically semi-autonomous, capable of encoding genes related to their function, and involved in the regulation of key metabolic processes such as cell differentiation, apoptosis, growth, and division [3]. The first land plant mitochondrial genome, that of *Marchantia polymorpha*, was determined in 1992 [4], followed by the *Arabidopsis thaliana* genome in 1997 [5]. With advances in sequencing technology, the study of mitochondrial genomes has expanded exponentially, revealing several key characteristics: there is significant size variation among different species [6]; mitochondrial genomes exhibit complex and variable structures, such as linear, circular, and branched forms [7,8]; despite variations in base composition, sequence, and copy number, 24 core gene types are consistently present across species, indicating a high level of conservation [9]; mitochondrial genomes contain abundant non-coding regions and a high frequency of RNA editing events [10]; and mitochondrial genomes evolve at slower rates compared to nuclear and chloroplast genes [11]. Given these characteristics and advantages, mitochondrial genomes are increasingly being used for family, genus, and higher-level classification research. They play a vital role in plant molecular identification and systematic classification studies.

In the genus *Gentiana*, the mitochondrial genomes of *G. crassicaulis* and *G. straminea* have been previously reported [12], with studies confirming that fragments of the mitochondrial genome contain stable variants suitable for species identification [13]. As *G. rigescens* is one of the primary species within the genus *Gentiana*, it possesses significant scientific research value. Understanding its mitochondrial genome is crucial for providing new insights into its evolutionary history and enriching the genetic data available.

This study aimed to assemble and annotate the *G. rigescens* mitochondrial genome, emphasizing its key characteristics and structural features. We analyzed relative synonymous codon usage (RSCU) and repeat sequences, while also considering the potential transfer of chloroplast DNA into the mitogenome. Furthermore, we investigated RNA editing sites in protein-coding genes (PCGs) and examined their phylogenetic relationships. The results offer fresh insights into the mitochondrial evolution of *Gentiana* plants and lay the groundwork for the development of genetic resources and molecular marker-assisted breeding in *G. rigescens*.

## 2. Results

### 2.1. Mitochondrial Structure and Gene Content of G. rigescens

**Figure 1:**
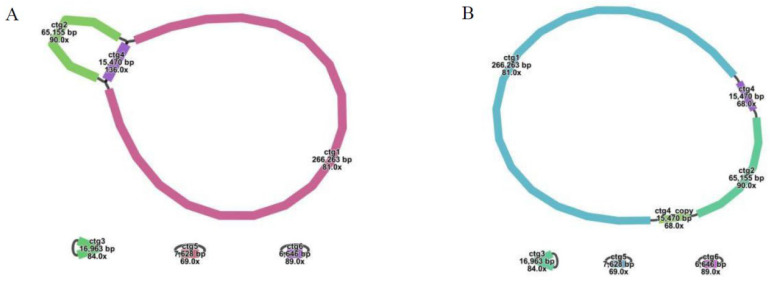
Mitochondrial genome assembly diagram, with node ID marked in the diagram. (**A**) Mitochondrial genome assembly primitive diagram. (**B**) Mitochondrial genome assembly final simplified diagram.

**Figure 2:**
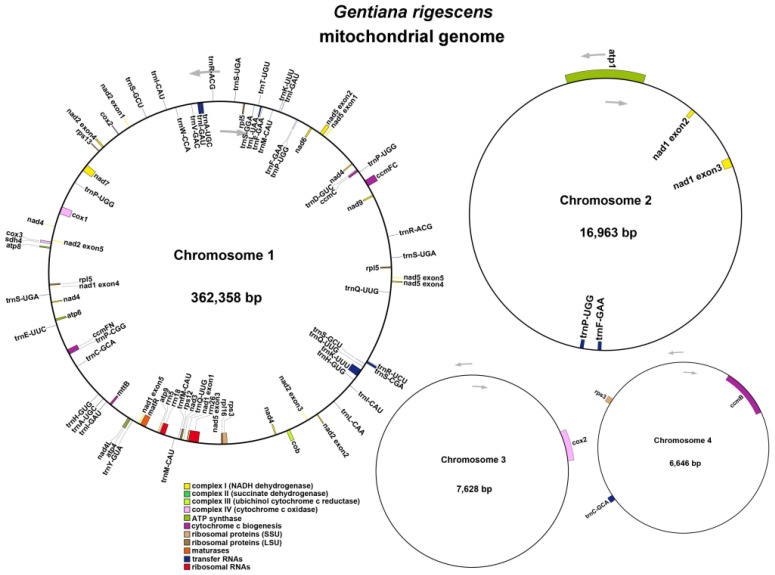
Mitochondrial genome characterization of *G. rigescens*. Different colors indicate various functional groups.

The mitochondrial genome map was constructed utilizing long-read sequencing data, encompassing several circular genomic DNA sequences, as depicted in Figure 1A. In the case of the multi-branch structure composed of ctg1, ctg2, and ctg4, we streamlined it into a linear sequence following the trajectory of ctg1-ctg4-ctg2-ctg4_copy, as illustrated in Figure 1B. Details of each node in the assembly are presented in Table 1. Consequently, we identified four distinct circular chromosomes with lengths of 6646 bp, 7628 bp, 16,963 bp, and 362,358 bp (accession numbers PQ124156, PQ124157, PQ124158, and PQ124159, respectively). The GC content for these chromosomes was determined to be 43.61%, 45.95%, 44.51%, and 43.64%, respectively. The cumulative length of the mitochondrial genome was 393,595 bp, with an overall GC content of 43.73% (Figure 2).

A total of 30 unique PCGs were annotated in our study, which included 24 mitochondrial core genes and 6 non-core genes. Additionally, we identified 26 tRNA genes, with 13 having multiple copies, and 3 rRNA genes. The core genes consisted of five ATP synthase genes (*atp*1, *atp*4, *atp*6, *atp*8, and *atp*9), nine NADH dehydrogenase genes (*nad*1, *nad*2, *nad*3, *nad*4, *nad*4L, *nad*5, *nad*6, *nad*7, and *nad*9), four cytochrome C biogenesis genes (*ccm*B, *ccm*C, *ccm*FC, and *ccm*FN), and three cytochrome C oxidase genes (*cox*1, *cox*2, and *cox*3). Also included were one membrane transport protein gene (*mtt*B), one matase gene (*mat*R), and one pantothenol–cytochrome C reductase gene (*cob*). The non-core genes encompassed two ribosomal large subunit genes (*rpl*5, *rpl*16), three ribosomal small subunit genes (*rps*3, *rps*12, *rps*13), and a succinate dehydrogenase gene (*sdh*4), as detailed in Table 2.

### 2.2. Codon Usage Analysis of PCGs

**Figure 3:**
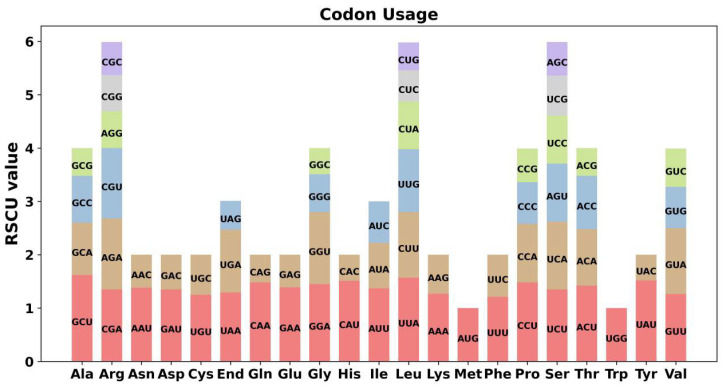
Codon usage bias of mitochondrial PCGs of *G. rigescens*.

The codon preference of 30 unique PCGs was analyzed. Codons with relative synonymous codon usage (RSCU) greater than 1 were considered to be used by amino acid preference. Moreover, the RSCU values for the start codon AUG and UGG (Trp) were both 1. Additionally, mitochondrial PCGs exhibited a general codon usage bias. Specifically, codons for GCU (Ala), CAA (Gln), CAU (His), UUA (Leu) and UAU (Tyr) showed a higher usage bias (Figure 3).

### 2.3. Repeat Elements

**Figure 4:**
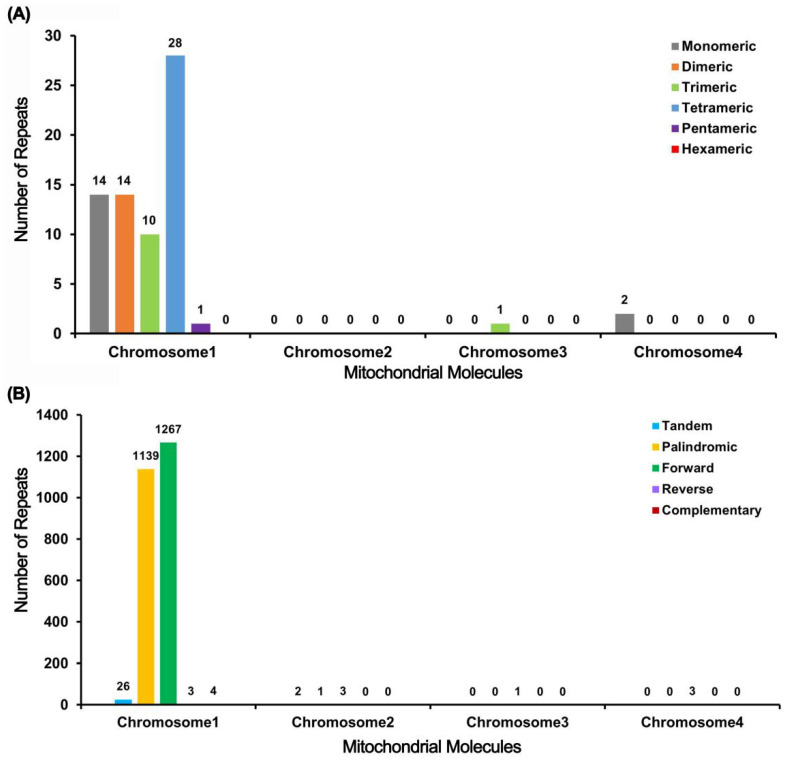
Repeat sequence analysis histogram. The abscissa represents the mitochondrial molecule and the ordinate represents the number of repeated fragments. (**A**) Grey legend represents monomeric SSRs, orange legend represents dimeric SSRs, green legend represents trimeric SSRs, blue legend represents tetrameric SSRs, purple legend represents pentameric SSRs, and red legend represents hexameric SSRs. (**B**) Blue legend represents tandem repeats, yellow legend represents palindromic repeats, green legend represents forward repeats, purple legend represents reverse repeats, and red legend represents complementary repeats.

A total of 70 SSRs were found in the mitochondrial genome, and most of them were found in Chromosome 1, with a total of 67 SSRs, of which tetrameric SSRs accounted for the largest proportion, with a total of 28 SSRs accounting for 41.79 %. It is worth noting that in 14 monomer SSRs, adenine (A) monomer repeats 12, accounting for 85.71% of monomeric SSRs. Scattered repeats were identified in Chromosome 1, including 26 tandems and 2413 pairs of repeats with lengths of 30 or more. This total included 1139 pairs of palindromes, 1267 pairs of forwards, 3 pairs of reverses, and 4 pairs of complementarities. (Figure 4).

### 2.4. Plastid DNA Insertion in Mitogenome

**Figure 5:**
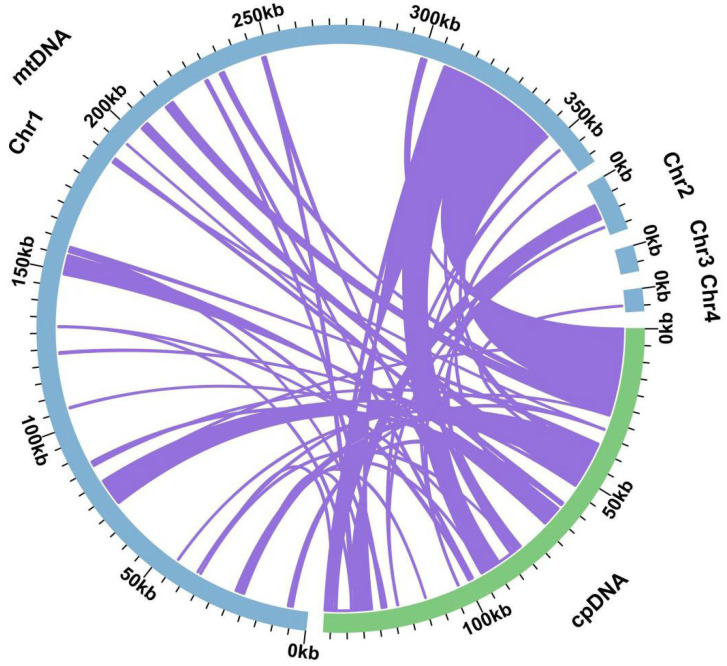
Analysis of gene transfer between the mitochondrial and chloroplast genomes of *G. rigescens*. The purple lines connecting the arcs indicate the homologous fragments shared between the two genomes.

Sequence similarity analysis revealed that 48 fragments in the species *G. rigescens* were homologous to both the mitochondrial and chloroplast genomes, with a combined length of 93,385 bp. This represented 23.73% of the total length of the mitochondrial genome (cpDNA: PQ137794). These fragments were numbered according to their length, each prefixed with “MTPT”. The longest fragment, MTPT21, spanned 23,330 bp, which was 5.93% of the mitochondrial genome’s total length (Figure 5).

By annotating these homologous sequences, 33 complete genes were found on 48 homologous fragments, including 21 PCGs (*atp*A, *atp*F, *atp*H, *atp*I, *clp*P, *mat*K, *ndh*B, *psa*J, *psb*A, *psb*I, *psb*K, *rpl*2, *rpl*20, *rpl*23, *rpl*33, *rpo*C1, *rpo*C2, *rps*18, *rps*7, *ycf*2, *ycf*15) and 11 tRNA genes (*trn*D-GUC, *trn*G-UCC, *trn*H-GUG, *trn*I-CAU, *trn*K-UUU, *trn*L-CAA, *trn*P-UGG, *trn*Q-UUG, *trn*R-UCU, *trn*S-GCU, *trn*W-CCA) (Supplementary Table S1; Supplementary Figure S1).

### 2.5. Synteny and Phylogenetic Analysis

**Figure 6:**
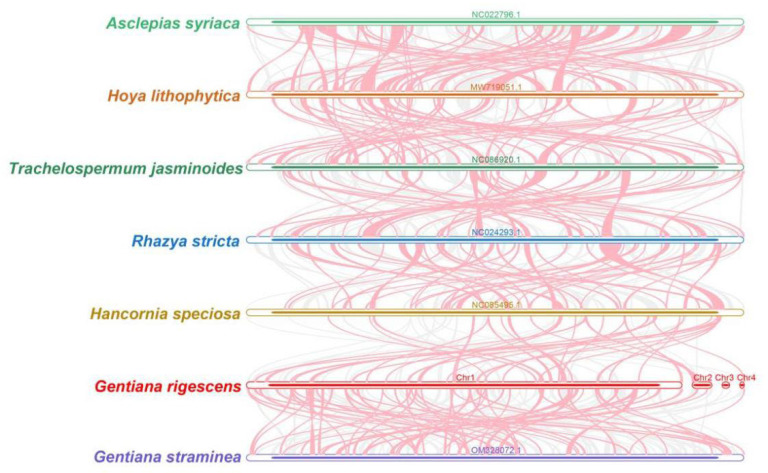
Synteny analysis of 7 mitogenomes.

Figure 6 illustrates the comparison between the mitochondrial genomes of *G. rigescens* and related species in Gentianales. The red arc region indicates an inverted segment, while the gray region signifies a well-conserved homologous region. For clarity, collinear blocks shorter than 0.5 kb were excluded from the analysis. Despite detecting homologous collinear blocks, they were notably short. Additionally, some regions were found to be unique to *G. rigescens*, showing no homology with other species.

The analysis revealed inconsistencies in the order of collinear blocks across the mitochondrial genomes of the seven species studied. This suggests that the mitochondrial genome of *G. rigescens* has undergone significant genome rearrangements compared to its related species. The sequences’ arrangement was highly variable, indicating a lack of conservation in the mitochondrial genome structure among these species.

Phylogenetic trees for 31 angiosperm species across four orders were constructed using DNA sequences from 24 conserved mitochondrial PCGs. These common genes included *atp*1, *atp*4, *atp*6, *atp*8, *atp*9, *ccm*B, *ccm*C, *ccm*FC, *ccm*FN, *cob*, *cox*2, *cox*3, *mat*R, *mtt*B, *nad*1, *nad*2, *nad*3, *nad*4, *nad*4L, *nad*5, *nad*6, *rps*3, *rps*12, and *rps*13. Two mitochondrial genomes from the order Azalea were designated as outgroups.

**Figure 7:**
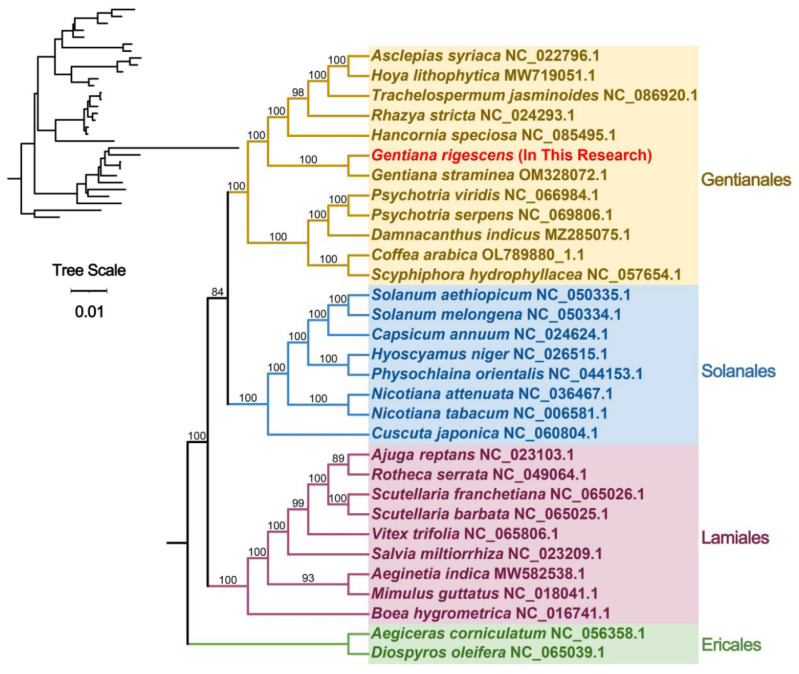
Phylogenetic tree constructed from the shared mitochondrial PCGs of 24 species.

The resulting phylogenetic topology, based on mitochondrial DNA, aligned with the most recent classification by APG (Angiosperm Phylogeny Group). *G. rigescens* was confirmed to be part of the *Gentiana* genus and was closely clustered with *G. straminea (*Figure 7).

### 2.6. RNA Editing Events

The RNA editing events of 30 unique PCGs from *G. rigescens* mitochondria were identified. A total of 393 latents were identified. The RNA editing sites were all base C to U editing. Among the mitochondrial genes, the *ccm*B gene identified 34 RNA editing sites, with the highest number of editing. Followed by *nad*4, 30 RNA editing sites were identified.

**Figure 8:**
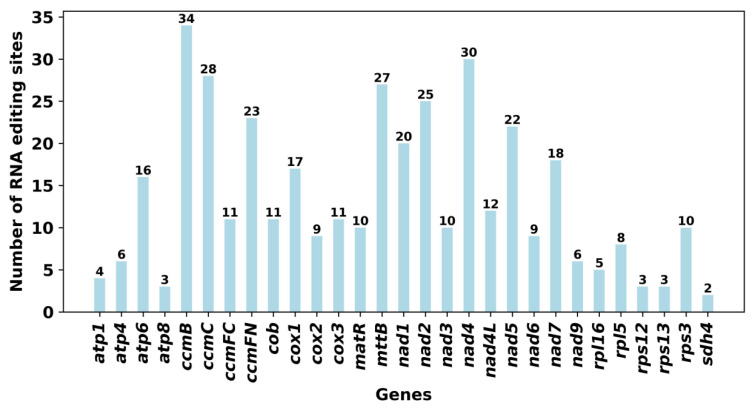
The number of RNA editing sites predicted by each mitochondrial PCGs.

We found that the start codons of 18 genes (*atp*1, *atp*4, *atp*6, *ccm*B, *ccm*C, *ccm*FC, *ccm*FN, *cox*1, *cox*2, *cox*3, *mtt*B, *nad*1, *nad*2, *nad*4, *rpl*5, *rps*12, *rps*13, *sdh*4) were generated by RNA editing events. Most of the start codons were from UCG to UUG, and the amino acids had Ser to Leu, or CCG to CUG, and Pro to Leu, which were supported by the high confidence of Deepred-mt (Supplementary Table S2) (Figure 8).

## 3. Discussion

The size of mitochondrial genomes exhibits significant variation among plant species. For instance, the mitochondrial genome of *Viscum scurruloideum* is only 66 kb in size [14], whereas *Larix sibirica* has the largest mitochondrial genome size, with 11.7 Mb [15]. The mitochondrial genome of *G. rigescens* is 393,595 bp (medium size). Plant mitochondrial genomes are characterized by their intricate combinations, diverse structural forms, and dynamic non-coding regions, which include highly recombinant repetitive sequences. The functionality of these genomes is largely dictated by their structural features [7]. The study of plant mitochondrial genomes reveals a remarkable diversity in chromosome structures even within the same family, underscoring the evolutionary adaptability and complexity of these genomes. For instance, species such as *G. straminea* [12], *Asclepias syriaca* [16], and *Rhazya stricta* [17] exhibit typical circular chromosomes. In contrast, *G. rigescens* features a main ring chromosome accompanied by three smaller rings. The mitochondrial genome of *Paphiopedilum micranthum* is unique, consisting of 26 circular chromosomes [18]. Furthermore, the *Quercus acutissima* genome is composed of a single linear molecule and two circular molecules [19]. This structural diversity is indicative of the high adaptability of mitochondrial genomes, enabling them to respond effectively to a variety of cellular and environmental conditions.

The GC content can indicate the stability and complexity of a DNA sequence [20,21]. In our study, we found that the mitochondrial genome of *G. rigescens* is 393,595 bp in size, with a GC content of 43.73%. Compared to the mitogenomes of *G. straminea* (410,086 bp, 44.55%) and *G. crassicaulis* (368,808 bp, 44.83%), the GC content is quite similar, which may represent the general GC content of the *Gentiana* mitochondrial genome. Although there are significant differences in mitochondrial genome sizes across species, the genes are relatively conserved. In the phylogenetic analysis, only *G. rigescens* and *G. straminea* clustered on the same branch, while *G. crassicaulis* did not, further suggesting that the mitochondrial genome may be suitable for higher-resolution taxonomic studies.

In higher plants, the number, location, and order of mitochondrial protein-coding genes may vary among different species and even among varieties within the same species, but there is often a remarkable consistency in gene repertoire [22]. For instance, across 21 species of Poaceae, there is a preference for 25 to 30 codons. There is also a notable similarity in the usage of the third base among mitochondrial coding gene sequences in different Poaceae species. This pattern of codon usage could be attributed to the conservative nature of codon usage bias in the mitochondrial genomes of Poaceae species during evolution [23]. Similarly, *G. rigescens* exhibits a significant number of preferred codons. Specifically, 30 codons show a higher usage preference, including GCU (Ala), CAA (Gln), CAU (His), UUA (Leu), and UAU (Tyr), which likely reflects a characteristic of *G. rigescens* shaped by long-term evolutionary selection. However, further studies are needed to identify any specific rules governing this codon preference.

Repeat sequences are widely present in the mitochondrial genome, including tandem, short palindrome, and large repeat sequences. The repeat sequence is one of the sources of mitochondrial gene rearrangement, which can directly mediate recombination [24,25,26]. Li [26] reported a pair of direct repeats (175 bp) with mediated recombination in the mitotic genome of *Scutellaria tsinyunensis*, dividing the 354,073 bp main loop into two chromosomes that are 25,5741 bp and 98,402 bp in length. According to the traditional multivariate theory, large repeats in the main sequence induce intragenomic recombination. Through long-read sequencing, eight structural paths for the *Opuntia dillenii* mitochondrial genome were identified, confirming the presence of multiple mitochondrial genome conformations. The authors selected the structure supported by most long-read sequences as the primary focus for analysis. In this structure, two direct repeat sequences were identified and mapped onto the assembly, revealing the main conformation of the cactus mitochondrial genome, which consists of a primary circular structure [27]. In our study, the structural path was relatively simple; however, a pair of repeat sequences, ctg4 and ctg4_copy, caused the merging of ctg2, ctg4_copy, ctg4, and ctg1 into a primary ring, forming a multi-ring structure in the *G. rigescens* mitochondrial genome, which consists of one primary ring and three smaller rings. At the same time, mostly palindromic repeats and forward repeat sequences were detected. It is worth noting that only 70 SSRs were detected, which was less than for other species, such as 165 SSRs in *Opuntia dillenii* Hance [27], 213 SSRs in *G. crassicaulis*, and 250 SSRs in *G. straminea* [12], and the conditions for developing molecular markers were limited.

The mitochondrial genome contains heterozygous sequences that may come from plastids, nuclei, and even other species [28,29]. Comparatively, in *Angelica dahurica* [30], *G. straminea*, and *G. crassicaulis* [12], these MTPTs constitute a mere 0.76%, 13.42%, and 12.61%, respectively, while, in *G. rigescens,* these MTPTs account for 23.73% of the total length of the mitochondrial genome. In particular, MTPT21 was the longest sequence, measuring 23,330 bp and accounting for 5.93% of the mitochondrial genome. Furthermore, 18 genes were annotated within this fragment. These may be an evolutionary feature of *G. rigescens,* which can provide new evidence for the evolution of plant mitochondrial genomes. Most notably, MTPT3, MTPT10, MTPT11, MTPT12, MTPT24, MTPT25, and MTPT38 are located in the inverted repeat region of the chloroplast genome, resulting in repeats of these sequences in the chloroplast. These sites are considered to be key hotspots for sequence migration, indicating that they play an important role in genomic structural dynamics.

RNA editing, a post-transcriptional modification, is prevalent in higher plants and plays a crucial role in mitochondrial gene expression, influencing plant physiology and molecular function [31]. Typically, RNA editing in angiosperms involves a site-specific C to U deamination reaction. In *G. rigescens*, a comprehensive analysis identified 393 potential RNA editing sites, with the majority located in the first or second position of codons, aligning with the patterns observed in most plants [32,33,34]. Identifying these sites allows for the effective prediction of gene function under the new codon context. It is worth noting that the start codons of 18 genes were created by RNA editing events and were highly trusted by Deepred-mt. The incorporation of these novel codons during translation modifies the amino acid composition, specifically increasing the presence of Leu. This alteration could potentially enhance mitochondrial gene expression and, consequently, improve the environmental adaptability of *G. rigescens*.

## 4. Materials and Methods

### 4.1. Plant Sampling, DNA Extraction, and Sequencing

Fresh leaf samples of *G. rigescens* were collected from Lincang, Yunnan, China, for the isolation of chloroplast and mitochondrial genomes. Total genomic DNA was extracted using the cetyltrimethylammonium bromide (CTAB) method [35]. Subsequently, Nanopore and Illumina sequencing were performed by Wuhan Bena Technology Co., Ltd.

### 4.2. Mitochondrial Genome Assembly and Annotation

Firstly, the *G. rigescens* mitochondrial genome was assembled based on long-read data. The long-read sequencing data were directly assembled using the default parameters of Flye [36] software to obtain graphical assembly results in GFA format. For all the assembled fasta format contigs, we used makeblastdb to build the library and then used the BLASTn program to identify the contig fragments containing the mitochondrial genome, using the mitochondrial gene in Arabidopsis as the query sequence. The parameter was “-evalue 1e-5-outfmt 6-max_hsps 10-word_size 7-task blastn-short”. Bandage software (v0.8.1) [37] was used to visualize the GFA file, the contigs of mitochondria were screened according to the results of BLASTn, and the mitochondrial genome sketch of species *G. rigescens* was obtained (SRA: PRJNA1142854).

Secondly, we used bwa software (v0.7.17) [38] to compare long-read and short-read data to mitochondrial contigs, filtered and exported the reads of the mitochondria in the comparison, and saved them for subsequent mixed assembly. At the same time, in order to solve the repetitive regions in the obtained graphical genome, bwa software was used to compare long reads from mitochondria to repetitive sequences to determine the path supported by more long reads to solve the repetitive regions. The same method was used to determine the most likely genomic structure of the mitochondrial genome for the remaining contigs containing multiple adaptors.

Finally, combined with the sequencing data of the above short-reads and long-reads, the *G. rigescens* mitochondrial genome was assembled using a hybrid assembly strategy. We used the default parameters of Unicycler [39] to achieve hybrid assembly and finally obtain the *G. rigescens* mitochondrial genome. The mitochondrial genome was visualized using Bandage v0.8.1.

Annotation method of mitochondrial genome: Protein-coding genes of mitochondrial genomes *Arabidopsis thaliana* (NC_037304), *G. straminea* (OM328072), and *Liriodendron tulipifera* (NC_021152.1) were selected as reference genomes, and the genome of the mitochondrial genome was annotated by Geseq v2.03 [40]. In addition, we also used the angiosperm mitochondrial genome annotation tool IPMGA (http://www.1kmpg.cn/ipmga/, accessed on 1 August 2024) to annotate the species. IPMGA has a good effect on the annotation of splice sites and trans-splicing genes. The tRNA of the mitochondrial genome was annotated using tRNAscan-SE v.2.0.11 [41]. The rRNA of the mitochondrial genome was annotated using BLASTN v2.13.0 [42]. The annotation errors of each mitochondrial genome were manually corrected using Apollo v1.11.8 [43].

### 4.3. Analysis of Codon Usage

The protein-coding sequence of the genome was extracted using Phylosuite v1.1.16 [44]. The codon bias of protein-coding genes in the mitochondrial genome was analyzed by MEGA v7.0 [45], and the RSCU value was calculated.

### 4.4. Analysis of Repeat Elements

MISA v2.1 (https://webblast.ipk-gatersleben.de/misa/, accessed on 1 August 2024) [46], TRF (v4.09) (https://tandem.bu.edu/trf/trf.unix.help.html, accessed on 1 August 2024) [47], and the REPuter network server (https://bibiserv.cebitec.uni-bielefeld.de/reputer/, accessed on 1 August 2024) [48] were used to identify repeats including microsatellite sequence repeats, tandem repeats, and scattered repeats, respectively. The results were visualized using Excel (2021) software and the Circos package (v0.69.9) [49].

### 4.5. Analysis of Chloroplast DNA Insertion in Mitogenome

The chloroplast genome was assembled using GetOrganelle [50], the chloroplast genome was annotated using CPGAVAS2 [51], and then the annotation results of the chloroplast genome were corrected using CPGView [52]. Homologous fragments were analyzed using BLASTN v2.13.0 [42] and the results were visualized using the Circos package (v0.69.9) [49].

### 4.6. Synteny and Phylogenetic Analysis

The BLASTn program was used to identify conserved homologous sequences, called collinear blocks, with parameters of “-value 1e-5, -word _ size 9, -gapopen 5, -gapextend 2, -reward 2, -penalty-3”. Only collinear blocks with a length of more than 500 bp were selected for further analysis. Using MCscanX [53], a pairwise comparison of multiple synteny plots was generated based on the results obtained by BLASTn order, and a conservative collinearity block was drawn.

In order to explain the genetic relationship between *G. rigescens* and other species, the phylogenetic tree based on the maximum likelihood (ML) method was constructed in PhyloSuite v1.1.16 [44], MAFFT v7.505 [54], and IQ-TREE v1.6.12 (the parameter: “-alrt 1000, -B 1000”) [55]. The phylogenetic tree was displayed in iTOL [56].

### 4.7. Analysis of RNA Editing Events

To better understand the RNA editing events in the mitochondrial genome of *G. rigescens*, we used 30 PCGs for analysis. To predict the RNA editing sites, C was converted to U in Deepred-mt [57]. This tool is based on a convolutional neural network (CNN) model for prediction. All results with probability values greater than 0.9 were retained.

## 5. Conclusions

In this study, we unveiled the mitogenome characteristics of the Yunnan specialty medicinal plant, *G. rigescens*. The mitogenome genome of *G. rigescens* consists of four circular subgenomes and has a length of 393,595 bp. We compared this with two medicinal plants of the same family and genus (*G. straminea* and *G. crassicaulis*). Our study enriches the mitochondrial genome database of *Gentiana* and lays a foundation for the study of the *Gentiana* mitochondrial genome.

## Figures and Tables

**Table 1:** The length and sequencing depth of each node.

Node	Length (bp)	Depth (×)
ctg1	266,263	81
ctg2	65,155	90
ctg3	16,963	84
ctg4	15,470	136
ctg5	7628	69
ctg6	6646	89

**Table 2:** Mitochondrial genome coding genes.

Group of Genes	Name of Genes
ATP synthase	*atp*1, *a*tp4, *at*p6, *at*p8, *atp*9
NADH dehydrogenase	*n*a*d*1, *nad*2, *nad*3, *nad*4, *nad*4L, *nad*5, *nad*6, *nad*7, *nad*9
Cytochrome *b*	*cob*
Cytochrome *c* biogenesis	*ccm*B, *ccm*C, *ccm*FC, *ccm*FN
Cytochrome c oxidase	*cox*1, *cox*2, *c*ox3
Maturases	*mat*R
Protein transport subunit	*mtt*B
Ribosomal protein large subunit	*rpl*5(×3), *rpl*16
Ribosomal protein small subunit	*rps*3, *rps*12, *rps*13
Succinate dehydrogenase	*sdh*4
Ribosome RNA	*rrn*5, *rrn*18, *rrn*26
Transfer RNA	*trn*A-UGC(×2), *trn*C-GCA(×2), *trn*D-GUC, *trn*E-UUC, *trn*F-GAA(×3), *trnf*M-CAU, *trn*H-GUG(×2), *trn*I-CAU(×2), *trn*I-GAU(×3), *trn*KUUU(×2), *trn*L-CAA, *trn*L-UAA, *trn*M-CAU(×2), *trn*P-CGG, *trn*P-UGG(×4), *trn*Q-UUG(×3), *trn*R-ACG(×2), *trn*R-UCU, *trn*S-CGA, *trn*S-GCU(×2), *trn*S-GGA, *trn*S-UGA(×3), *trn*T-UGU, *trn*V-GAC, *trn*W-CCA, *trn*Y-GUA

## Data Availability

Data Availability Statement: The mitochondrial genome sequence has been registered on NCBI (https://www.ncbi.nlm.nih.gov, accessed on 1 August 2024) and the accession numbers are PQ124156, PQ124157, PQ124158, and PQ124159. The samples are deposited in the Laboratory of Molecular Biology, College of Traditional Chinese Medicine, Yunnan University of Traditional Chinese Medicine, with the voucher number 20231116NL1-02.

## Supplementary Materials

The following supporting information can be downloaded at: https://www.mdpi.com/article/10.3390/ijms252111428/s1.

## References

[1] National Pharmacopoeia Commission (2020). Pharmacopoeia of the People’s Republic of China: 2020 Edition.

[2] Picault N., Hodges M., Palmieri L., Palmieri F. (2004). The growing family of mitochondrial carriers in *Arabidopsis*. Trends Plant Sci..

[3] Dyall S.D., Brown M.T., Johnson P.J. (2004). Ancient invasions: From endosymbionts to organelles. Science.

[4] Takemura M., Oda K., Yamato K., Ohta E., Nakamura Y., Nozato N., Akashi K., Ohyama K. (1992). Gene clusters for ribosomal proteins in the mitochondrial genome of a liverwort, *Marchantia polymorpha*. Nucleic Acids Res..

[5] Unseld M., Marienfeld J.R., Brandt P., Brennicke A. (1997). The mitochondrial genome of *Arabidopsis thaliana* contains 57 genes in 366,924 nucleotides. Nat. Genet..

[6] Wynn E.L., Christensen A.C. (2018). Repeats of Unusual Size in Plant Mitochondrial Genomes: Identification, Incidence and Evolution. G3 Genes Genomes Genet..

[7] Kozik A., Rowan B.A., Lavelle D., Berke L., Schranz M.E., Michelmore R.W., Christensen A.C. (2019). The alternative reality of plant mitochondrial DNA: One ring does not rule them all. PLoS Genet..

[8] Varré J.S., D’Agostino N., Touzet P., Gallina S., Tamburino R., Cantarella C., Ubrig E., Cardi T., Drouard L., Gualberto J.M. (2019). Complete Sequence, Multichromosomal Architecture and Transcriptome Analysis of the *Solanum tuberosum* Mitochondrial Genome. Int. J. Mol. Sci..

[9] Mower J.P. (2020). Variation in protein gene and intron content among land plant mitogenomes. Mitochondrion.

[10] Lukeš J., Kaur B., Speijer D., Dave S. (2021). RNA Editing in Mitochondria and Plastids: Weird and Widespread. Trends Genet..

[11] Qiu Y.-L., Li L., Wang B., Xue J.-Y., Hendry T.A., Li R.-Q., Brown J.W., Liu Y., Hudson G.T., Chen Z.-D. (2010). Angiosperm phylogeny inferred from sequences of four mitochondrial genes. J. Syst. Evol..

[12] Ala K., Zhao Z., Ni L., Wang Z. (2023). Comparative analysis of mitochondrial genomes of two alpine medicinal plants of *Gentiana* (Gentianaceae). PLoS ONE.

[13] Lu J.N., Zhou Z.L., Ni L.H., Gaawe D., Mi M. (2019). The identification of Sect. Cruciata (*Gentiana*) species using mtDNA nad1/b-c and nad5/d-e fragments. Acta Pharm. Sin..

[14] Skippington E., Barkman T.J., Rice D.W., Palmer J.D. (2015). Miniaturized mitogenome of the parasitic plant *Viscum scurruloideum* is extremely divergent and dynamic and has lost all nad genes. Proc. Natl. Acad. Sci. USA.

[15] Putintseva Y.A., Bondar E.I., Simonov E.P., Sharov V.V., Oreshkova N.V., Kuzmin D.A., Konstantinov Y.M., Shmakov V.N., Belkov V.I., Sadovsky M.G. (2020). Siberian larch (*Larix sibirica* Ledeb.) mitochondrial genome assembled using both short and long nucleotide sequence reads is currently the largest known mitogenome. BMC Genom..

[16] Straub S.C., Cronn R.C., Edwards C., Fishbein M., Liston A. (2013). Horizontal transfer of DNA from the mitochondrial to the plastid genome and its subsequent evolution in *milkweeds* (apocynaceae). Genome Biol. Evol..

[17] Park S., Ruhlman T.A., Sabir J.S., Mutwakil M.H., Baeshen M.N., Sabir M.J., Baeshen N.A., Jansen R.K. (2014). Complete sequences of organelle genomes from the medicinal plant *Rhazya stricta* (Apocynaceae) and contrasting patterns of mitochondrial genome evolution across asterids. BMC Genom..

[18] Yang J.X., Nicolas D., Bai M.Z., Guo Y.Y. (2023). Multichromosomal Mitochondrial Genome of *Paphiopedilum micranthum*: Compact and Fragmented Genome, and Rampant Intracellular Gene Transfer. Int. J. Mol. Sci..

[19] Liu D., Guo H.L., Zhu J.L., Qu K., Chen Y., Guo Y.T., Ding P., Yang H.P., Xu T., Jing Q. (2022). Complex Physical Structure of Complete Mitochondrial Genome of *Quercus acutissima* (Fagaceae): A Significant Energy Plant. Genes.

[20] Zhang X.F., Jacob B.L., Wang H.X., Zhi-Xin Zhu Z.X., Wang H.F. (2021). Comparative analysis of chloroplast genome structure and molecular dating in *Myrtales*. BMC Plant Biol..

[21] Mahajan S., Agashe D. (2022). Evolutionary jumps in bacterial GC content. G3 Genes Genomes Genet..

[22] Parvathy S.T., Udayasuriyan V., Bhadana V. (2021). Codon usage bias. Mol. Biol. Rep..

[23] Yang S.Q., Liu J.F., Jiang S.C., Guo B.Q., Guo J., Cao L.L., Zhang W. (2024). Mitochondrial genome characteristics and phylogenetic analysis of 21 species of Poaceae. Genom. Appl. Biol..

[24] Yu R., Sun C., Zhong Y., Liu Y., Sanchez-Puerta M.V., Mower J.P., Zhou R. (2021). The minicircular and extremely heteroplasmic mitogenome of the holoparasitic plant *Rhopalocnemis phalloides*. Curr. Biol..

[25] Dong S.S., Zhao C.X., Chen F., Liu Y.H., Zhang S.Z., Wu H., Zhang L.S., Liu Y. (2018). The complete mitochondrial genome of the early flowering plant *Nymphaea colorata* is highly repetitive with low recombination. BMC Genom..

[26] Li J.L., Xu Y.C., Shan Y.Y., Pei X.Y., Yong S.Y., Liu C., Yu J. (2021). Assembly of the complete mitochondrial genome of an endemic plant, *Scutellaria tsinyunensis*, revealed the existence of two conformations generated by a repeat-mediated recombination. Planta.

[27] Zhang X., Shan Y.Y., Li J.L., Qin Q.L., Yu J., Deng H.P. (2023). Assembly of the Complete Mitochondrial Genome of *Pereskia aculeata* Revealed That Two Pairs of Repetitive Elements Mediated the Recombination of the Genome. Int. J. Mol. Sci..

[28] Chun Y.H., Nicole G., Nahal A., Jeremy N.T., William M. (2005). Mutational decay and age of chloroplast and mitochondrial genomes transferred recently to angiosperm nuclear chromosomes. Plant Physiol..

[29] Turmel M., Otis C., Lemieux C. (2002). The chloroplast and mitochondrial genome sequences of the charophyte *Chaetosphaeridium globosum*: Insights into the timing of the events that restructured organelle DNAs within the green algal lineage that led to land plants. Proc. Natl. Acad. Sci. USA.

[30] Li Y.Y., Liu Y.Y., Zeng X., Wu P., Li Q.M., Guo S.X., Hao Z.G. (2024). Complete mitochondrial genome of *Angelica dahurica* and its implications on evolutionary analysis of complex mitochondrial genome architecture in Apiaceae. Front. Plant Sci..

[31] Small I.D., Schallenberg-Rüdinger M., Takenaka M., Mireau H., Ostersetzer-Biran O. (2019). Plant organellar RNA editing: What 30 years of research has revealed. Plant J..

[32] Bi C.W., Lu N., Xu Y.Q., He C.P., Lu Z.H. (2020). Characterization and Analysis of the Mitochondrial Genome of Common Bean (*Phaseolus vulgaris*) by Comparative Genomic Approaches. Int. J. Mol. Sci..

[33] Yang H.X., Li W.H., Yu X.L., Zhang X.Y., Zhang Z.Y., Liu Y.X., Wang W.X., Tian X.X. (2021). Insights into molecular structure, genome evolution and phylogenetic implication through mitochondrial genome sequence of *Gleditsia sinensis*. Sci. Rep..

[34] Grewe F., Edger P.P., Keren I., Sultan L., Pires J.C., Ostersetzer-Biran O., Mower J.P. (2014). Comparative analysis of 11 Brassicales mitochondrial genomes and the mitochondrial transcriptome of *Brassica oleracea*. Mitochondrion.

[35] Aboul-Maaty N.A.-F., Oraby H.A.-S. (2019). Extraction of high-quality genomic DNA from different plant orders applying a modified CTAB-based method. Bull. Natl. Res. Cent..

[36] Kolmogorov M., Yuan J., Lin Y., Pevzner P.A. (2019). Assembly of long, error-prone reads using repeat graphs. Nat. Biotechnol..

[37] Wick R.R., Schultz M.B., Zobel J., Holt K.E. (2015). Bandage: Interactive visualization of de novo genome assemblies. Bioinformatics.

[38] Heng L., Richard D. (2010). Fast and accurate long-read alignment with Burrows-Wheeler transform. Bioinformatics.

[39] Wick R.R., Judd L.M., Gorrie C.L., Holt K.E. (2017). Unicycler: Resolving bacterial genome assemblies from short and long sequencing reads. PLoS Comput. Biol..

[40] Tillich M., Lehwark P., Pellizzer T., Ulbricht-Jones E.S., Fischer A., Bock R., Greiner S. (2017). GeSeq- versatile and accurate annotation of organelle genomes. Nucleic Acids Res..

[41] Lowe T.M., Eddy S.R. (1997). tRNAscan-SE: A program for improved detection of transfer RNA genes in genomic sequence. Nucleic Acids Res..

[42] Chen Y., Ye W.C., Zhang Y.D., Xu Y.S. (2015). High speed BLASTN: An accelerated MegaBLAST search tool. Nucleic Acids Res..

[43] Lewis S.E., Searle S.M., Harris N., Gibson M., Lyer V., Richter J., Wiel C., Bayraktaroglu L., Birney E., Crosby M.A. (2002). Apollo: A sequence annotation editor. Genome Biol..

[44] Zhang D., Gao F.L., Jakovlić I., Zou H., Zhang J., Li W.X., Wang G.T. (2019). PhyloSuite: An integrated and scalable desktop platform for streamlined molecular sequence data management and evolutionary phylogenetics studies. Mol. Ecol. Resour..

[45] Kumar S., Stecher G., Tamura K. (2016). MEGA7: Molecular Evolutionary Genetics Analysis Version 7.0 for Bigger Datasets. Mol. Biol. Evol..

[46] Beier S., Thiel T., Münch T., Scholz U., Mascher M. (2017). MISA-web: A web server for microsatellite prediction. Bioinformatics.

[47] Benson G. (1999). Tandem repeats finder: A program to analyze DNA sequences. Nucleic Acids Res..

[48] Kurtz S., Choudhuri J.V., Ohlebusch E., Schleiermacher C., Stoye J., Giegerich R. (2001). REPuter: The manifold applications of repeat analysis on a genomic scale. Nucleic Acids Res..

[49] Zhang H.G., Meltzer P., Davis S. (2013). RCircos: An R package for Circos 2D track plots. BMC Bioinform..

[50] Jin J.J., Yu W.B., Yang J.B., Song Y., dePamphilis C.W., Yi T.S., Li D.Z. (2020). GetOrganelle: A fast and versatile toolkit for accurate de novo assembly of organelle genomes. Genome Biol..

[51] Shi L.C., Chen H.M., Jiang M., Wang L.Q., Wu X., Huang L.F., Liu C. (2019). CPGAVAS2, an integrated plastome sequence annotator and analyzer. Nucleic Acids Res..

[52] Liu S.Y., Ni Y., Li J.L., Zhang X.Y., Yang H.Y., Chen H.M., Liu C. (2023). CPGView: A package for visualizing detailed chloroplast genome structures. Mol. Ecol. Resour..

[53] Wang Y.P., Tang H.B., Debarry J.D., Tan X., Li J.P., Wang X.Y., Lee T.H., Jin H.Z., Marler B., Guo H. (2012). A toolkit for detection and evolutionary analysis of gene synteny and collinearity. Nucleic Acids Res..

[54] Katoh K., Standley D.M. (2013). MAFFT multiple sequence alignment software version 7: Improvements in performance and usability. Mol. Biol. Evol..

[55] Nguyen L.T., Schmidt H.A., von Haeseler A., Minh B.Q. (2015). IQ-TREE: A fast and effective stochastic algorithm for estimating maximum-likelihood phylogenies. Mol. Biol. Evol..

[56] Letunic I., Bork P. (2019). Interactive Tree Of Life (iTOL) v4: Recent updates and new developments. Nucleic Acids Res..

[57] Edera A.A., Small I., Milone D.H., Sanchez-Puerta M.V. (2021). Deepred-Mt: Deep representation learning for predicting C-to-U RNA editing in plant mitochondria. Comput. Biol. Med..

