# Supplementary figures and images for: Mitochondrial Genome Assembly and Structural Characteristics Analysis of Gentiana rigescens
Source: Int J Mol Sci. 2024 Oct 24;25(21):11428. doi: 10.3390/ijms252111428 (PMC11546909; doi:10.3390/ijms252111428)

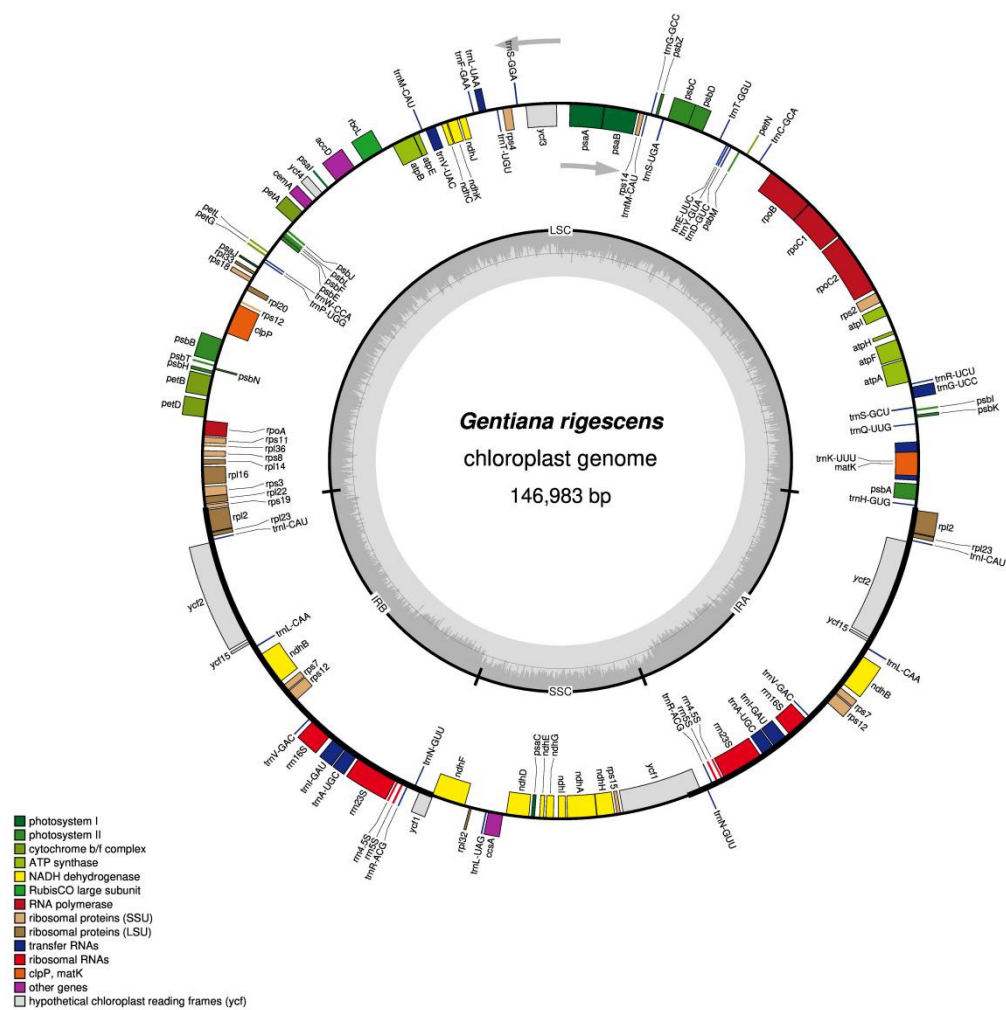

Supplementary Figures S1:cpDNA maps of *G. rigescens*.

Supplement: Supplementary file 1 [file ijms-25-11428-s001.zip › Supplementary Figures.pdf]
